# Mucormycosis and COVID-19: Double trouble for India?

**DOI:** 10.7189/jogh.12-03001

**Published:** 2022-02-12

**Authors:** Farah Yasmin, Hala Najeeb, Sarush Ahmed Siddiqui, Rohan Kumar Ochani

**Affiliations:** Department of Internal Medicine, Dow Medical College, Dow University of Health Sciences, Karachi, Pakistan

The Coronavirus disease-19 (COVID-19) pandemic caused by the severe acute respiratory syndrome coronavirus-2 (SARS-CoV-2) initially emerged as a pneumonia-like illness in the city of Wuhan, China in December 2019. The deadly virus owing to its rapid transmissibility rate spread like a wildfire across international borders and was soon declared as a matter of global emergency by the World Health Organization in March 2020. Since then, this highly infectious virus, possessing the capability to strangle an individual on a ventilator has claimed over three million lives worldwide [1]. The advent of many kinds of COVID-19 vaccines has curtailed serious outbreaks and has been proven to reduce COVID-19 related mortality and hospitalizations.

However, developing countries like Bangladesh, Nepal, and Pakistan scrambled to secure vaccines for the vulnerable population while India, overestimated its COVID-19 vaccine production and began exporting millions of doses [2]. Religious ceremonies, political rallies, and the eased lockdown by the country which believed it was on its way to defeat the virus witnessed a record high tally of 414 000 active cases by May 6 during the deadly third wave of the COVID-19 pandemic [3]. The crippled Indian health care system has run out of hospital beds, oxygen cylinders, ventilators, and COVID-19 therapy treatments, which it received from countries worldwide. Individuals are forced to fight for their lives at home, and without any medical assistance, often succumb to death. The highly transmissible Indian strain (B 1.167) has an expanding spectrum of symptoms, ranging from the common cardiopulmonary manifestations to the recently emerging vesicular lesions, urticaria, and rashes [1,4].

## PATHOGENESIS AND CLINICAL MANIFESTATIONS OF MUCORMYCOSIS IN COVID-19 PATIENTS

As health care professionals continue to struggle to flatten the curve, frequent cases of a rare invasive fungal infection, mucormycosis, have emerged all over India since the first-ever reported case in December 2020. Mucormycosis, caused by the Mucorales fungi, is present as spores in a hot and humid environment, and in the soil as compost and rotting produce. This opportunistic infection typically leads to nasal and sinus congestion, and infection of the jawbone along with neutropenia [5]. Though non-transmissible among individuals and animals, inhaling spores or an infection from a traumatic skin lesion leads to rhino-orbital-cerebral, pulmonary, gastrointestinal, cutaneous, renal, and disseminated mucormycosis [6]. The most commonly occurring rhino-orbital-cerebral mucormycosis affects the paranasal sinuses, and eyes. It presents as nasal congestion and discharge, facial pain, or swelling among other signs. This barbaric form of the disease can extend into the orbit, lead to diplopia (double vision), redness of eyes, and eventual blindness [5,6]. This particular type necrotizes vascular tissues, hence is incorrectly referred to as the Black Fungus [7]. Pulmonary mucor, the second common form, involves the lungs. It presents as hemoptysis, patchy infiltrates, and consolidation which are revealed on chest CT scans.

**Figure:**
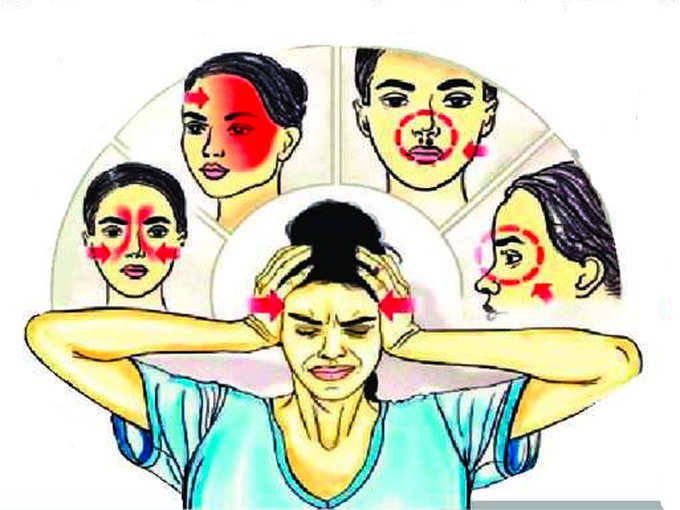
Photo: The picture, from https://digitalhayat.in/, depicts the entry, and early manifestations of mucormycosis. Spores are inhaled and damage the orbits after passing through sinuses. This leads to the initial symptoms of headache and numbness.

The SARS-CoV-2 affects the upper respiratory tract via angiotensin-converting enzyme 2 (ACE2) receptors, leading to pneumonia, vascular, endothelial damage, and reduced CD4+ and CD8+ T cell count, thus weakening the immune system, thereby making an individual highly susceptible to bacterial and fungal infections [7]. The national and international reports of the perpetually increasing incidence of COVID-19-associated mucormycosis in recovered or active patients all over the country have led to health care professionals fearing an endemic alongside a pandemic [8].

This life-threatening disease, if left untreated, has a mortality of 45%-90% in immunocompromised individuals having an ICU stay of >50 days, and in those with co-morbidities such as cancer, ischemic heart disease, chronic kidney disease, organ transplants, and diabetes. Uncontrolled diabetes, especially diabetic ketoacidosis remains the leading predisposing risk factor of mucormycosis in India which has 77 million diabetic adults [6]. The deadly trinity – COVID-19, hyperglycemia and mucormycosis release a cytokine cascade that delays IFN-γ release and puts the diabetic individual at a greater risk of developing endotheliitis and hence, mucormycosis in several organs like the heart, liver, and kidney [9].

## IDENTIFYING CAUSES OF THE MUCORMYCOSIS OUTBREAK

COVID-19 patients worldwide have been undergoing therapy with nuclear analogue antivirals such as Remdesivir which received emergency approval in multiple countries. Dexamethasone, a glucocorticoid, Chloroquine, and Hydroxychloroquine proved significant in controlling COVID-19 in hospitalized patients. However, the drug trials either included a small COVID-19 sample or had their trials terminated. Azithromycin has reduced mortality but has shown adverse cardiac effects [10]. To control the exponentially rising COVID-related mortality rate in India, physicians have been extraordinarily recommending steroids. India’s population is prone to practicing polypharmacy, as much as 93% in Uttaranchal [11]. Contributing to excessive steroid use is the easy availability of over-the-counter medicines in pharmacies across India. Such immunosuppressants elevate glucose levels and alter anti-inflammatory metabolism, providing favourable conditions for the Mucorales fungi.

Exhausted health care facilities and the reduced professional help have instilled a fear of losing a life outside a hospital. Thus, most people have turned to self-medication, including administering oxygen mechanically. However, regular cleaning of oxygen masks, humidifiers, and the maintenance of oxygen cylinders and ventilators is required in hospital settings or otherwise. Tap water, boiled water, and unfiltered water are used instead of distilled water in cylinders which exasperates the development of fungi in pipes and endangers lives [12]. Moreover, the approaching monsoon season in southern India with its humid weather can be a possible threat to the daily count of mucormycosis.

As COVID-19 plagued India, the people of Gujrat, Delhi, Haryana, and Odisha turned to cow-dung therapy. The belief that covering themselves in cow-dung and urine weekly will provide immunity and recovery from COVID-19 stems from a deeply rooted spiritual and religious association with the sacred cow. Cow shelters are breeding grounds for mucoromycetes and health care professionals hypothesize this to be a weapon of mass destruction for the already-suffering nation [13].

## PREVENTING AND MANAGING MUCORMYCOSIS AND COVID-19 COINFECTION

Thus, controlling risk factors such as neutropenia, hyperglycaemia, and ketoacidosis through close monitoring of blood glucose levels in COVID-19 active and recovered patients is the best preventive strategy. Staging and grading techniques of Mucoromycetes along with appropriately measured dosage recommendations of steroids and antifungals can help health care workers all over the country to fight this growing plague. Mucormycosis that often appears as black patches around the nasal region can be immediately detected by testing the patch with potassium permanganate solution [12]. First-line treatment of mucormycosis includes controlling previously stated risk factors, and the use of Liposomal Amphotericin B (antifungal) [5,7]. Fosmanogepix, a first-class antifungal drug to treat invasive fungal infections, has its trial in the pipeline [14]. Non-contrast computed tomography remains the first choice of investigation of paranasal sinus infection followed by magnetic resonance imaging (MRI). Necrotizing tissue should immediately be removed through extensive surgical debridement to prevent the spread. It is essential that non-facility-based patients are continually monitoring to detect the initial signs to prevent organ rotting ie, loss of eyes, removal of the jawbone, or necrotized brain tissue [5].

Indian Council of Medical Research recommends the use of a peripherally inserted central catheter (PICC) to administer normal saline before antifungals. As hospital services remain burdened, home services for mucormycosis diagnostic tests should be made available. Imposing a strict lockdown, as followed by countries such as China and New Zealand, and practising self-discipline will control the self-destructive COVID and mucormycosis emergency. In the age of broad-spectrum antibiotics and steroids, scientists and researchers need to be wary of further fungal or bacterial outbreaks. The lack of authentic information on COVID-19 and foreseeable outbreaks should be highlighted through social media engagement. Medical graduates can contribute to in-house care through telemedicine, conveying medically authentic information through applications or calls. Antifungals for mucormycosis are expensive and unaffordable for the public in India. Thus, public service messages by the government should focus on the prevention of the disease, rather than treatment as highlighting names of antifungal medicines leads to illegal black marketing.

An emerging endemic that has already infected 211 individuals and claimed 16 lives in Aurangabad [15], has crossed borders affecting 18 countries including India’s immediate neighbour, Pakistan [16]. Pakistan has recently reported its first mucormycosis cases, four of which succumbed to death [17]. Albeit the unfavourable conditions and medical advances worldwide, governments and health ministries must prepare for outbreaks immediately.
